# Soap-Suds

**Published:** 1869-03

**Authors:** 


### SOAP-SUDS

Is an excellent fertilizer of grass and grape-vines, and should not be wasted. A bit of alum in a tub of soap-suds sends the dirt and soap to the bottom and leaves the water fit for use again, if carefully poured off from the sediment, which becomes a concentrated fertilizer, almost as good as guano. If a lump of alum as large as the thumb-joint is thrown into four or five gallons of boiling soap-suds, the scum runs over and leaves the water clean and soft and useful for washing. We have often, in ancient times, "settled" a glass of Mississippi water, and made it look as "clear as a bell" in a few seconds by tying a bit of alum to a string and twirling it around under the surface of the water in the glass.

